# The effect of barberry (Berberis integerrima) on lipid profile and systemic inflammation in subjects with cardiovascular risk factors: a randomized controlled trial

**DOI:** 10.1186/s12906-022-03539-8

**Published:** 2022-03-07

**Authors:** Hadi Emamat, Ali Zahedmehr, Sanaz Asadian, Javad Nasrollahzadeh

**Affiliations:** 1 Department of Clinical Nutrition and Dietetics, Faculty of Nutrition Sciences and Food Technology, National Nutrition and Food Technology Research Institute, Shahid Beheshti University of Medical Sciences, Tehran, Iran; 2 Rajaie Cardiovascular Medical and Research Center, Iran University of Medical Sciences, Tehran, Iran

**Keywords:** Barberry, Lipid profile, Inflammation, CVD

## Abstract

**Background:**

Despite significant advances in the management of cardiovascular disease (CVDs), there is still a large burden of CVD in the world. The inclusion of functional foods in the diet may provide beneficial effects on CVD. Purple-black barberry due to its richness in anthocyanins and berberine has shown beneficial effects on cardiometabolic factors. We investigated the effects of barberry on plasma lipids as well as inflammatory biomarkers in subjects with cardiovascular risk factors.

**Methods:**

This was an 8-weeks, single-blinded, randomized controlled clinical trial that the participants were randomly assigned to a barberry (10 g/day dried barberry) or placebo group. At baseline and end of the study, plasma lipid profiles including total cholesterol (TC), high-density lipoprotein cholesterol (HDL-C), low-density lipoprotein cholesterol (LDL-C), triglycerides (TG), small-dense LDL-C (sd-LDL-C), non-HDL-C, and TC/HDL-C, as well as inflammatory biomarkers including C-reactive protein (CRP) and interleukin-6 (IL-6), were determined. An intention-to-treat analysis was performed.

**Results:**

Eighty-four participants were randomly assigned to study groups. The mean (± SD) participants' age was 54.06 ± 10.19 years. Body weight, body mass index (BMI), physical activity, and dietary intake were not different between the two groups at baseline and the end of the study. After adjusting for baseline values, we observed a significant decrease in plasma levels of TG, TC, LDL-C, sd-LDL-C, non-HDL-C, and TC/HDL-C (*p* < 0.001, *p* = 0.011, *p* = 0.015, *p* = 0.019, *p* = 0.004, and *p* = 0.039 respectively) as well as CRP (*p* = 0.020) in the barberry group compared to the placebo group.

**Conclusions:**

Our results indicate that purple-black barberry consumption decreases plasma levels of CRP and improves lipid profile in subjects with cardiovascular risk factors.

**Trial registration:**

This clinical trial was registered at ClinicalTrials.gov (NCT number: NCT04084847).

## Introduction

Despite significant advances in the prevention and treatment of cardiovascular disease (CVDs), there is still a large burden of CVD in many parts of the world [1]. Approximately one-third of global mortality is due to CVD that leading to a rise in health care costs [2, 3]. Various risk factors for CVDs have been identified, including diabetes, dyslipidemia, and hypertension [4]. Dyslipidemia, including an increase in plasma low-density lipoprotein cholesterol (LDL-C) levels, is a major risk factor for atherosclerosis [5]. In addition to the LDL-C levels, the size and density of LDL particles are important in the development and progression of atherosclerosis. Smaller LDL particles (sd-LDL) have a higher density and are more atherogenic. Smaller LDL particles penetrate the endothelium more easily, are more prone to oxidation, and have a greater ability to bind to vascular intima proteoglycans and produce more mediators of vasoconstriction and aggregation [6]. In addition to classical risk factors, newer risk factors such as inflammation and oxidative stress have also been proposed for CVD. Circulating levels of C-reactive protein (CRP) and interleukin-6 (IL-6) are known biomarkers for assessing the inflammatory status in the body that are involved in the formation of atherosclerotic plaques and their rupture [7].

Lifestyle modifications, including dietary factors, play an important role in the prevention of CVD [8], and the inclusion of functional foods in the diet may provide further beneficial effects [9]. Berries are polyphenol-rich foods that have beneficial effects on the control of CVDs risk factors [10, 11]. Anthocyanins which are abundant in berries [12] have been shown to be effective in the prevention of CVD by improving inflammatory processes, as well as dyslipidemia [13]. Purple-black barberry (berberis integerrima), is a berry fruit with a sharp flavor and is considered an herbal medicine [14]. Barberry due to its richness in anthocyanins and berberine has shown beneficial effects on cardiometabolic factors in experimental studies [15, 16]. In vitro evidence has shown that barberry extract or its biologically active compounds can suppress lipid peroxidation without having a cytotoxic effect on normal human blood cells [17]. The effects of barberry on inflammation, plasma lipid profile, and blood pressure have been studied in animal models [18–23]. Barberry extract at doses of 25 and 100 mg/kg body weight has reduced fasting blood sugar, triglycerides (TG), lipid oxidation and lipoprotein (a) in diabetic rats [20]. Furthermore, an antihypertensive effect of barberry extract has been observed in rats [23]. The effects of barberry consumption on plasma lipid concentrations and inflammatory biomarkers have been investigated in limited clinical trials, however the results have been partly inconsistent. [17, 24–27]. In patients with metabolic syndrome, 600 mg/day of dried barberry for 6 weeks reduced plasma CRP but had no significant effect on LDL-C levels [24].

Given that limited clinical trials have evaluated the effects of barberry on plasma lipid and inflammatory markers, and the observed results have not been consistent, our aim in the present study was to investigate the effects of barberry on plasma lipids including TG, LDL-C, sd-LDL-C, and HDL-C as well as inflammatory biomarkers including CRP and IL-6 in subjects with cardiovascular risk factors.

## Material and methods

### Study design

The protocol of the present trial has previously been described [25]. This was an 8-weeks, single-blinded, parallel assigned, randomized controlled clinical trial (RCT) that the participants were kept ignorant of either the group to which they have been assigned, but the investigator who assigned patients to intervention or placebo groups was not blinded. Patients between the ages of 20 and 65 years with a history of hypertension who were treated with stable doses of antihypertensive drugs prior to the study, and had at least one other classical CVD risk factor, including diabetes mellitus and/or hyperlipidemia were recruited from an academic hospital clinic (Rajaei Cardiovascular, Medical, and Research Center, Tehran, Iran) who periodically referred to hospital clinics. The reluctance to continue interventions, any changes in the medication or surgical treatment during the study, BMI > 30 kg/m^2^, taking nitrate or high doses of statin drugs (atorvastatin > 40 mg/day or rosuvastatin > 20 mg/day), regular intake of vitamin or mineral supplements in the last month, and chronic kidney disease stage 4 or 5 were the exclusion criteria. The participants had the option to leave the study at any time. The study was approved by the Ethics Committee of National Nutrition and Food Technology Research Institute, Shahid Beheshti University of Medical Sciences, Tehran, Iran. The ethical committee code was IR.SBMU.NNFTRI.REC.1398.003. The study protocol was according to the Helsinki Declaration. Informed consent was obtained from the participants. This clinical trial was registered at http://www.ClincalTrials.gov, identifier: NCT04084847, on 10/09/2019.

In the first meeting, the aim and method of the study were described to the participants and informed consent was obtained from them. Participants were then entered into the 2-week maintenance weight program (run-in) and were asked not to change their lifestyle. In the next visit (baseline) participants were randomized to receive either the barberry or the placebo product. The randomization was performed with the aid of a sequence generated by using a random number table. For this purpose, each row of the table was considered as a block, and from the left, in the order in the table, odd numbers were considered as group A and even numbers were considered as group B. If the first two allocations were for one of the A or B, the next two allocations were considered for the second group. The sequence of each block was placed in a sealed opaque envelope. To achieve a balanced distribution of diabetes and dyslipidemia in each of the study groups, stratified block randomization was used, with four strata: 1, with a history of diabetes and receiving statins; 2, with a history of diabetes and not receiving statins; 3, without a history of diabetes and receiving statins; 4, without a history of diabetes and not receiving statins. A block size of 4 within each stratum was used, with a 1:1 randomization between the two groups. The nutritional composition of dried barberry used in this study was determined by standard methods in a reference food laboratory (Table 1). The barberry powder contained 10 g milled dried purple-black barberry plus 1 g of milled sucrose and the placebo was a combination of 9 g maltodextrin, 1 g citric acid, 1 g milled sucrose plus edible red color. The barberry or placebo powders were packed delivered to the participants. To ensure that the participants adhered to the consumption of the packages, they were called twice a week. It was also determined to bring empty packages to the hospital at the end of the study and deliver them to the study coordinators. Participants were asked not to change the current level and type of physical activity as well as their eating habits during the study.

**Table 1:** Nutritional composition of dried barberry fruit

**Compounds**	**Value**
Carbohydrate (g/ 100 g)^a^	49.8
Protein (g/ 100 g)^a^	7.7
Fat (g/ 100 g)^a^	3.2
Fiber (g/ 100 g)^a^	23.8
Total polyphenol (mg /ml of extract) ^b^	12.19
Moisture (%)^a^	8.7

^a^Determined in the dried fruit sample

^b^Determined in an ethanolic extract of dried barberry fruit (by the Folin-Ciocalteu reagent) and were expressed as mg gallic acid equivalent per ml of extract obtained from 1 g of dried barberry

General information)including anthropometrics, demographic, disease history, medications, smoking status, and physical activity), were obtained by a questionnaire. Habitual dietary intake was collected using three 24-h dietary recall questionnaires (2 regular workdays and 1 day at the weekend) through face-to-face and telephone interviews at baseline and at week-8 of the study and was analyzed using software (Nutritionist 4, First Data Bank, San Bruno, CA, USA). Furthermore, adapted dietary inflammatory index (DII) was determined from 36 food items that were available in the participants’ dietary composition analysis [26].

A 10 ml blood sample was taken after 12 h fasting state at baseline and the end of the study, and plasma was separated by centrifugation (4000 rpm for 20 min) and samples were stored at − 80 °C. All measurements of each of the studied parameters were analyzed in batch and together (baseline samples along with end-of-study samples) to minimize inter-assay variation. Plasma lipid profiles including TC, HDL-C, and TG were measured by an enzymatic method (colorimetry) using Pars Azmoon kits (Pars-Azmoon, Karaj, Iran) and autoanalyzer (Selectra ProXL, Vital Scientific, Spankeren, The Netherlands). LDL-C concentration was calculated using the Friedwald formula. To measure sd-LDL, first lipoproteins with a density of less than 1.044 g/mL were precipitated using a heparin-magnesium precipitating solution, then LDL-C was measured in clear surface liquid by colorimetric method using a direct LDL-C measuring kit [27]. Plasma levels of CRP were measured with a latex microparticle-enhanced immunoassay kit (Audit Diagnostics, Cork, Ireland) and using an autoanalyzer. The IL-6 level was measured using a commercial enzyme-linked immunosorbent assay kit (Biolegend, San Diego, USA).

### Statistical analysis

In the original study, plasma lipids and inflammatory biomarkers were considered as secondary outcomes and the sample size was determined based upon systolic blood pressure as the primary outcome variable, by considering a pooled value of the standard deviation = 8.8, with type I error (α) of 0.05, power of 0.80, and an attrition rate of 20%, a total of 80 participants (40 in each group) were regarded as the study sample size. Results were analyzed by SPSS software (version 21.0, SPSS Inc., Chicago, IL). All analyses were conducted using intention-to-treat and the baseline carry forward method was used to manage missing data. The data normality determination was assessed by Shapiro–Wilk test. The mean ± standard deviation (SD) and frequency (percent) were used for the quantitative and the qualitative data presentation, respectively. Between-group comparisons for and qualitative and quantitative data were performed using Chi-Square (χ2), and analysis of covariance (ANCOVA) adjusted for baseline levels, respectively. Within-group comparisons were done by paired t-test.

## Results

**Fig. 1:**
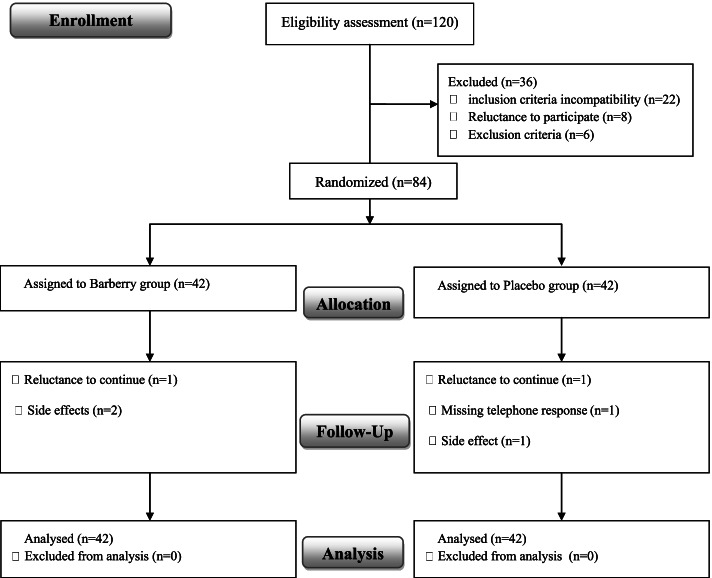
CONSORT flowchart

Of the 84 participants who were randomly assigned to study groups and 78 participants completed the study (39 in barberry and 39 in the placebo group) (Fig. 1) and six were excluded from the study (2 cases of refusing to continue, 1 case of missing telephone response, and 3 cases of side effects that two of the participants in the barberry group reported mild diarrhea, and one in the placebo group reported bloating). Patient compliance was good throughout the study, and the subjects consumed more than 80% of the packages delivered to them.

Baseline characteristics of the participants including age, gender, CVD risk factors including diabetes, dyslipidemia, and smoking were similar between barberry and placebo groups (Table 2). The mean participants' age was 54.06 ± 10.19 years. Forty-five percent of subjects were male, 67.9% had dyslipidemia, 32.1% were smokers, 33.3% had diabetes and all of them had hypertension and were being treated with antihypertensive drugs. In addition, participants' body weight, body mass index (BMI), and physical activity were not statistically different between the two groups at baseline and end of the study (Table 2).

**Table 2:** Characteristics of the participants

Characteristic		Barberry group (*n* = 42)	Control group (*n* = 42)	*P*-Value^a^
Age (years)		53.62 ± 10.34	54.50 ± 10.13	0.69
Sex (male)		22 (52.4%)	16 (38.1%)	0.18
Diabetes		14 (33.3%)	14 (33.3%)	1
Dyslipidemia		30 (71.4%)	27 (64.2%)	0.64
Smoking		15 (35.7%)	12 (28.6%)	0.48
Statins drugs use				0.98
none		13 (30.9%)	13 (30.9%)	
Rosuvastatin 5 or Atorvastatin 10		4 (9.5%)	3 (7.1%)	
Rosuvastatin 10 or Atorvastatin 20		11 (26.1%)	11 (26.1%)	
Rosuvastatin 20 or Atorvastatin 40		12 (28.5%)	13 (30.9%)	
Weight (Kg)	Before	79.91 ± 13.52	75.42 ± 10.05	0.08
	After 2-month	79.71 ± 13.27	75.46 ± 9.93	0.10
BMI (Kg/m^2^)	Before	28.21 ± 2.03	27.83 ± 2.32	0.42
	After 2-month	28.16 ± 1.96	27.85 ± 2.30	0.51
Phisical activity (MET-hr/d)	Before	27.0 ± 3.85	26.61 ± 3.62	0.64
	After 2-month	27.38 ± 3.95	26.76 ± 3.90	0.47

Values are mean ± SD or n (%)

^a^Based on independent t-test or Chi-Square (χ2) test

The dietary intakes of the two groups are presented in Table 3. Dietary intake of energy and nutrients, as calculated from the 3-day dietary recall, was similar in two groups before and after 8 weeks follow-up (Table 3). There was no difference in the DII score of background diet between the two groups before and after the intervention.

**Table 3:** Dietary intake of the participants at the baseline and after 8 weeks

Variable	Time	Barberry group^a^ (*n* = 42)	Control group^a^ (*n* = 42)	*P*-value^b^
Energy (Kcal)	Baseline	2917.31 ± 233.59	2882.14 ± 277.82	0.53
	After 8 weeks	2871.12 ± 266.99	2864.43 ± 273.34	0.91
Carbohydrate (g)	Baseline	338.55 ± 68.73	357.15 ± 58.23	0.18
	After 8 weeks	335.42 ± 66.19	343.21 ± 50.77	0.54
Carbohydrate (%)	Baseline	46.39 ± 8.82	49.87 ± 8.59	0.07
	After 8 weeks	46.68 ± 8.20	48.30 ± 8.11	0.36
Protein (g)	Baseline	125.16 ± 40.07	125.71 ± 41.35	0.95
	After 8 weeks	120.55 ± 38.95	119.46 ± 36.56	0.89
Protein (%)	Baseline	17.14 ± 5.26	17.31 ± 5.07	0.88
	After 8 weeks	16.77 ± 5.27	16.52 ± 4.37	0.81
Total fat (g)	Baseline	123.87 ± 27.10	112.42 ± 26.63	0.06
	After 8 weeks	116.29 ± 26.49	112.57 ± 28.84	0.54
Total fat (%)	Baseline	38.22 ± 7.93	34.82 ± 6.37	0.06
	After 8 weeks	36.40 ± 7.51	35.09 ± 7.56	0.42
SFA (g)	Baseline	27.16 ± 6.98	25.65 ± 7.59	0.34
	After 8 weeks	26.61 ± 5.58	25.82 ± 5.38	0.51
SFA (%)	Baseline	8.44 ± 2.32	8.01 ± 2.20	0.39
	After 8 weeks	8.45 ± 2.03	8.16 ± 1.66	0.48
MUFA (g)	Baseline	31.30 ± 6.85	28.64 ± 6.92	0.08
	After 8 weeks	30.34 ± 5.71	28.76 ± 6.07	0.22
PUFA (g)	Baseline	52.07 ± 18.71	44.13 ± 16.47	0.06
	After 8 weeks	49.64 ± 17.41	43.54 ± 14.15	0.07
Omega-3 (mg)	Baseline	668.20 ± 499.11	601.30 ± 398.38	0.52
	After 8 weeks	659.50 ± 442.74	576.20 ± 352.18	0.47
Cholesterol (mg)	Baseline	463.86 ± 160.41	453.10 ± 179.24	0.77
	After 8 weeks	458.26 ± 165.82	450.73 ± 177.38	0.84
Fiber (g)	Baseline	28.06 ± 10.29	28.68 ± 9.21	0.77
	After 8 weeks	26.69 ± 9.16	27.91 ± 8.20	0.52
Sodium (mg)	Baseline	1413.18 ± 556.60	1577.44 ± 734.85	0.25
	After 8 weeks	1649.56 ± 769.70	1626.21 ± 590.92	0.87
DII score^a^	Baseline	0.55 ± 1.23	0.61 ± 1.26	0.82
	After 8 weeks	0.51 ± 1.17	0.63 ± 1.25	0.63

All values are mean ± SD

^a^Values are analyzed without taking into account the daily barberry or placebo consumption

^b^Data were compared using an independent t-test

Table 4 shows the measures of lipid profile in participants at the baseline and after 2 months in the barberry and placebo groups. There were no significant differences in baseline levels of TG, TC, LDL-C, HDL-C, and sd-LDL-C between the two groups. After adjusting for baseline values, between-group comparisons by ANCOVA revealed a significant decrease in plasma levels of TG (*p* < 0.001, power = 0.99), TC (*p *= 0.011, power = 0.73), LDL-C (*p* = 0.015, power = 0.68), sd-LDL-C (*p* = 0.019, power = 0.66), non-HDL-C (*p* = 0.004, power = 0.83) and TC/HDL-C (*p* = 0.039, power = 0.54) in the barberry group compared to the placebo group but there were no significant differences between the two groups regarding plasma HDL-C levels.

**Table 4:** Measures of lipid profile in participant at the baseline and after 8 weeks

Variable	Time	Barberry group (*n* = 42)	Control group (*n* = 42)	*p*-value^b^	*p*-value^c^
TC (mg/dL)	baseline	168.18 ± 35.74	180.92 ± 29.93	0.08	0.011
	after 8 weeks	160.23 ± 35.80	183.62 ± 31.52	0.002	
	*p*-value^a^	0.06	0.55		
LDL-C (mg/dL)	baseline	97.27 ± 36.22	107.64 ± 24.37	0.12	0.015
	after 8 weeks	89.72 ± 32.63	108.27 ± 25.45	0.005	
	*p*-value^a^	0.06	0.86		
sd-LDL (mg/dL)	baseline	33.07 ± 18.16	35.89 ± 18.70	0.48	0.019
	after 8 weeks	29.61 ± 15.82	39.87 ± 21.25	0.014	
	*p*-value^a^	0.24	0.25		
HDL-C (mg/dL)	baseline	44.41 ± 8.87	47.23 ± 9.31	0.15	0.21
	after 8 weeks	44.60 ± 10.70	45.23 ± 9.28	0.77	
	*p*-value^a^	0.87	0.022		
TC/HDL-C	baseline	3.88 ± 0.91	3.92 ± 0.75	0.80	0.039
	after 8 weeks	3.76 ± 1.18	4.17 ± 0.83	0.07	
	*p*-value^a^	0.44	0.023		
Non-HDL-C (mg/dL)	baseline	123.76 ± 33.20	133.69 ± 26.81	0.13	0.004
	after 8 weeks	115.58 ± 34.12	138.38 ± 29.45	0.002	
	*p*-value^a^	0.036	0.28		
TG (mg/dL)	baseline	131.23 ± 46.16	122.56 ± 43.58	0.37	< 0.001
	after 8 weeks	113.69 ± 32.86	150.56 ± 60.35	0.001	
	*p*-value^a^	0.002	< 0.001		

All values are means ± SD. *HDL-C*, high-density lipoprotein cholesterol; *LDL-C*, low-density lipoprotein cholesterol; *sd-LDL-C*, small-dense low-density lipoprotein cholesterol; *TG*, triglycerdes; *TC*, total cholesterol

^a^Values were compared using paired t-test

^b^Values were compared using an independent t-test

^c^Values were analyzed using the ANCOVA test with baseline values as a covariate

The baseline and end of study concentrations of plasma inflammatory markers in placebo and barberry groups are shown in Table 5. After adjusting for baseline values, plasma CRP levels decreased in the barberry group compared to the placebo group (*p* = 0.020, power = 0.64). A tendency to decrease in plasma IL-6 concentrations was observed in the barberry group compared to the placebo group. (*p* = 0.06, power = 0.49).

**Table 5:** Measures of inflammatory cytokines in participants at the baseline and after 8 weeks

Variable	Time	Barberry group (*n* = 42)	Control group (*n* = 42)	*p*-value^b^	*p*-value^c^
CRP (mg/L)	baseline	2.64 ± 1.28	2.34 ± 0.96	0.22	0.020
	after 8 weeks	1.95 ± 0.97	2.30 ± 0.98	0.10	
	*p*-value^a^	0.001	0.78		
IL-6 (pg/mL)	baseline	19.84 ± 10.54	20.38 ± 11.52	0.82	0.06
	after 8 weeks	19.50 ± 13.37	24.32 ± 13.75	0.10	
	*p*-value^a^	0.82	0.019		

All values are means ± SD. *CRP*, C-reactive protein; *IL-6*, Interleukin-6

^a^Values were compared using paired t-test

^b^Values were compared using an independent t-test

^c^Values were analyzed using the ANCOVA test with baseline values as a covariate

## Discussion

This study aimed to examine the effect of berberis integerrima consumption on lipid profile and systemic inflammation in subjects with cardiovascular risk factors. Our results showed that daily consumption of 10 g of the barberry powder for 8 weeks significantly improved inflammatory biomarker of CRP and plasma lipid profile except for HDL-C. There were no changes in participants' body weight, BMI, or diet during the study.

Several studies have examined the effects of barberry consumption on plasma lipid concentrations, but their results have been inconsistent [24, 28–31]. In the study by Shidfar et al., daily consumption of 3 g of barberry for 12 weeks in diabetic patients reduced total cholesterol, LDL-C, and TG but had no significant effect on HDL-C [30]. In contrast, Zilaee et al. reported that in patients with metabolic syndrome, 600 mg/day of barberry fruit extract for 6 weeks reduced total cholesterol, but had no significant effect on LDL-C or TG concentrations [24]. Ebrahimi et al. reported that in patients with type 2 diabetes, daily consumption of 5 g of barberry fruit with 770 mL of apple cider vinegar for 8 weeks was able to reduce LDL-C and increase HDL-C [25]. In the study of Lazavi et al., daily consumption of 200 ml of barberry juice in diabetic patients could only reduce total cholesterol [28]. Consistent with our findings in a meta-analysis study in 2019 that examined 5 clinical trials involving 339 participants, barberry supplementation significantly reduced total cholesterol (mean changes: -23.58 mg / dL), TG (mean changes: -29.16 mg / dL), and LDL-C (mean changes: -13.75 mg / dL) while it does not cause a significant change in HDL-C [28]. The contradictory results of these studies may be due to different designs, barberry form (as an extract, fruit, or juice), or the characteristics of the subjects (age, sex, health status, etc.).

Fruits, such as berries, contain polyphenol compounds that have been proposed to affect lipid metabolism in humans. Among the most important polyphenols in berries are anthocyanins, which are responsible for the red, blue, and purple colors of these fruits. Anthocyanins may reduce circulating LDL-C and TG [32]. Furthermore, berberine in barberry may also be one of the constituents to improve plasma lipid concentrations. Improving hepatic function and bile secretion, inhibiting cholesterol uptake in the intestine, and inhibiting the HMG-CoA reductase enzyme are proposed mechanisms in reducing cholesterol by berberine [30–32]. Berberine in barberry may also act as a ligand for PPAR-α [33], by which it may reduce plasma TG levels. In general, barberry reduces the expression of lipogenesis-related genes and increases the expression of genes related to energy consumption in muscle and adipose tissue [29]. There are also meta-analysis studies that show berberine as a predominant alkaloid in barberry can have beneficial effects on plasma lipids [34, 35]. Regarding the safe dose and side effects of berberine, a meta-analysis study reported that various studies have generally used doses between 0.6 and 2.7 g/day of berberine, and toxic reactions may occur in large doses. Most studies do not show serious side effects and the side effects are limited to mild gastrointestinal symptoms such as nausea, diarrhea, constipation, abdominal distension, and abdominal pain, which can be eliminated by reducing the dose of berberine to 0.6 g/day [35].

In the present study, barberry consumption reduced plasma CRP but had no significant effect on IL-6 concentration. A human study that examined the anti-inflammatory effect of barberry in patients with metabolic syndrome demonstrated that barberry could reduce CRP levels [17]. The lack of anti-inflammatory effects of barberry on IL-6 in this study may be related to sample size or duration of intervention. Consistent with our findings, a meta-analysis that examined the effects of the consumption of berries on CVD risk factors, showed that the consumption of berries results in a significant reduction of CRP and tumor necrosis factor-α (TNF-α) with no significant effect on IL-6 [10].

The anti-inflammatory effects of berries have been related to their antioxidant properties [33]. Anthocyanins are bioactive compounds present in berries that have antioxidant capability; however, their effects are not limited to their antioxidant properties and may affect specific steps in cell signaling pathways, including anti-inflammatory specific effects [36]. In addition to anthocyanins, several experimental and animal studies have shown the anti-inflammatory effects of berberine in barberry [37]. In an in vitro study by Lee et al. [38], berberine was able to reduce pro-inflammatory cytokines interleukin-1β and TNF-α in a dose-dependent manner in human lung cells. Inhibition of degradation of inhibitory kappaB-alpha that function to inhibit the nuclear factor kappa-B transcription factor was suggested as a mechanism for the suppressive effect of berberine on the inflammatory mediators' production.

This study had some strengths, such as good completion rates, its design, and good compliance with supplemental barberry. The data on some potential confounders such as weight, BMI, dietary intake, and level of physical activity of patients was evaluated. However, the present study had some limitations. Due to multiple eligibility criteria, the generalizability of the data is limited to hypertensive men and women being treated with specific medications. Furthermore, measurement of barberry phenolic compounds metabolites in urine or plasma could have provided useful information about the absorption and metabolism of consumed barberry polyphenols. Moreover, the study population was being treated with various drugs to control cardiovascular risk factors that have known side effects and also may reduce the effectiveness of barberry consumption on plasma lipids and inflammatory factors compared to people who do not take the drug.

## Conclusion

Our results indicate that seedless purple-black barberry consumption decreases plasma levels of CRP and improves lipid profile in subjects with cardiovascular risk factors. This improvement was independent of participants' weight, physical activity, and dietary intake. According to the results of this study, daily consumption of dried black barberry can be recommended to those who have CVD risk factors aiming to improve their lipid profile and inflammatory status. However, to confirm the results, further studies with a larger sample size and different amounts of barberry are recommended.

## Data Availability

The datasets generated and/or analyzed during the current study will be available from the corresponding author on reasonable request.

## Abbreviations

CVD: Cardiovascular disease
LDL-C: Low-density lipoprotein cholesterol
TC: Total cholesterol
HDL-C: High-density lipoprotein cholesterol
sd-LDL-C: Small-dense LDL-C
TG: Triglycerides
CRP: Creactive protein
IL-6: Interleukin-6
RCT: Randomized controlled clinical trial
DII: Dietary inflammatory index
ANCOVA: Analysis of covariance
BMI: Body mass index
TNF-α: Tumor necrosis factor-α
